# Gonadotropin Inhibitory Hormone and Its Receptor: Potential Key to the Integration and Coordination of Metabolic Status and Reproduction

**DOI:** 10.3389/fendo.2021.781543

**Published:** 2022-01-13

**Authors:** Grégoy Y. Bédécarrats, Charlene Hanlon, Kazuyoshi Tsutsui

**Affiliations:** ^1^ Department of Animal Biosciences, University of Guelph, Guelph, ON, Canada; ^2^ Graduate School of Integrated Sciences for Life, Hiroshima University, Higashihiroshima, Japan

**Keywords:** Gonadotropin inhibitory hormone (GnIH), RF-amide related peptide (RFRP), G-protein coupled receptor (GPCR), reproduction, metabolic control

## Abstract

Since its discovery as a novel gonadotropin inhibitory peptide in 2000, the central and peripheral roles played by gonadotropin-inhibiting hormone (GnIH) have been significantly expanded. This is highlighted by the wide distribution of its receptor (GnIH-R) within the brain and throughout multiple peripheral organs and tissues. Furthermore, as GnIH is part of the wider RF-amide peptides family, many orthologues have been characterized across vertebrate species, and due to the promiscuity between ligands and receptors within this family, confusion over the nomenclature and function has arisen. In this review, we intend to first clarify the nomenclature, prevalence, and distribution of the GnIH-Rs, and by reviewing specific localization and ligand availability, we propose an integrative role for GnIH in the coordination of reproductive and metabolic processes. Specifically, we propose that GnIH participates in the central regulation of feed intake while modulating the impact of thyroid hormones and the stress axis to allow active reproduction to proceed depending on the availability of resources. Furthermore, beyond the central nervous system, we also propose a peripheral role for GnIH in the control of glucose and lipid metabolism at the level of the liver, pancreas, and adipose tissue. Taken together, evidence from the literature strongly suggests that, in fact, the inhibitory effect of GnIH on the reproductive axis is based on the integration of environmental cues and internal metabolic status.

## Introduction

Following the initial discovery of gonadotropin-inhibiting hormone (**GnIH**) in quail over 20 years ago (1), homologues have been identified and characterized in multiple vertebrate species ranging from fish to mammals (for review: 2, 3). Structurally, GnIH and its homologues belong to the broader RF-amide peptides family with a unique LPXRFa (X=L or Q) C-terminal motif. As is the case for many novel peptides, GnIH was named after its reported inhibitory effects on gonadotropin release in quail. In fact, GnIH was the first hypothalamic peptide reported to exert an anti-gonadotrophic effect in any vertebrate species (1). Specifically, GnIH was shown to directly inhibit gonadotropin-releasing hormone (GnRH) release *via* the GnIH receptor (GnIH-R; 4), as well as downregulate luteinizing hormone beta-subunit (LHβ) mRNA levels (5) and inhibit its release (1) from the anterior pituitary gland. However, the role of GnIH on follicle-stimulating hormone beta-subunit (FSHβ) has been more ambiguous, as studies in quail have revealed no impact on mRNA levels (5) or FSH release (1), while both were suppressed in cultured cockerel pituitaries (6). Interestingly, although this effect was further confirmed in other avian species and extensively reviewed (7–11), it is less evident in mammals and remains controversial, especially as it relates to puberty (12). Beyond the reproductive axis, GnIH and its receptor have also been shown to participate in the control of energy homeostasis and nutrient partitioning through regulation of appetite control, glycemia, adipose, thyroid activity, and the stress response. Furthermore, since the GnIH-R in mammals is also activated by neuropeptide FF (NPFF), it has been shown to modulate nociception, although *via* activation by NPFF rather than GnIH (13). In this review, based on tissue distribution, cellular localization, and ligand availability, we explore the integrative neuroendocrine function of GnIH and its receptor to coordinate reproduction and energy homeostasis in response to multiple internal and external cues.

## Nomenclature of GnIH-R and its Ligands

Throughout the literature, while GnIH was originally named for its identified role in quail (1), orthologs in mammalian species are commonly annotated as LPXRFa peptides or RFamide-related peptides (RFRPs), specifically RFRP-3 (14). Additional annotations also used in the literature include neuropeptide VF (NPVF) and neuropeptide SF (NPSF) (15, 16). Similarly, receptors are often named after their known ligands, or when the ligand is unknown, based on the receptor type, genomic, and phylogenic information. In the case of RFamide peptides, this is further complicated by the promiscuity between receptors and ligands (for review: 17). As a result, non-avian GnIH-Rs are also referred to as Neuropeptide-FF receptor 1 (NPFF-R1) (13), receptor OT7T022 (15), and RFRP-R (7). In addition, based on the nomenclature of G protein-coupled receptors (GPR), the GnIH-R is known as GPR147 (18). In fact, despite the confusing nomenclature surrounding GnIH-Rs, it is now well accepted that the primary receptor for GnIH is GPR147, while another candidate, GPR74 (referred to as HLWAR77 or NPFF-R2), which is present in most vertebrates with the exception of fish (2, 19), displays a lower affinity for GnIH and may actually be more specific to NPFF (7, 8, 13, 16, 20). This preferential binding of NPFF to GPR74 also extends to the ligands neuropeptide AF (NPAF) and RFRP-1 (21). Thus, whenever possible for simplicity and coherence, we opted to refer to both ligand and receptors as GnIH and GnIH-Rs, respectively.

## GnIH-Rs Structure, Intracellular Signalling and Ligand Selectivity

To date, GnIH-Rs have been cloned or deduced from genomic databases across many vertebrate species (**Table 1**), including teleosts, aves, and mammals (for review: 2), and although most species possess a single GnIH-R, up to three paralogues have been reported in Goldfish (*Carassius auratus;* 49), Zebrafish (*Danio rerio;* 51), common carp (*Cyprinus Carpio*; 54) and more recently, the Indian Major Carp (*Labeo Catla)* in which GnIH-R paralogues were shown to belong to the GPR147 group, although forming their own subclade separate from mammalian and avian GPR147 (61). Interestingly, despite these differences in phylogeny, these GnIH-R paralogues have been reported to play similar roles in reproduction, as outlined throughout **Table 1**.

**Table 1:** List of GnIH-Rs orthologues across vertebrate species with localization and reported function.

MAMMALS								
Species	Sex^1^	Receptor		Ligand	Reported Function			References
		Name	Localization^2^		Reproductive	Metabolic	Other	
**Horse** (*Equus caballus*)	F	NPFFR-1	Hyp, Pit	RFRP-3	No effect on GnRH or LH release			(22)
**Syrian hamster** (*Mesocricetus auratus*)	M	GPR147	Hyp, BNST, HbN, Hpc	RFRP-3	Stimulates the HPG axis	Potential role as an intermediate between metabolic cues toward central reproductive control		(23)
	F	GPR147	Hyp, BNST, HbN, Hpc	RFRP-3	Inhibits LH release			
		GPR147	GnRH neurons, Kiss neurons	RFRP-3	Inhibits gonadotropin release in presence of GnRH stimulation			(24)
	F	GPR147	Pit	RFRP-3	Mediates LH surge at the level of the Pit			(25)
	M	GPR147	B, T	RFRP	Regulates spermatogenesis			(26)
**Siberian hamster** (*Phodopus sungorus*)	M	GPR147	GnRH neurons	RFRP-1	Inhibits LH release during LD; promotes LH release during SD; no effect on FSH			(27)
		GPR147	GnRH neurons	RFRP-3				
**Sheep **(*Ovis aries)*		NPFFR-1	SCN, PeVN, SON, PT	RFRP	Potential role in photoperiodic time measurement			(28)
**Human **(*Homo sapiens*)		GPR147	Adipose	NPFF		Slow antilipolytic effect		(29)
		GPR147	Adipose	NPSF		Rapid antilipolytic effect		
		GPR147	Ov	RFRP-3	Downregulates steroidogenesis			(30)
		GPR147	Hyp, Pit	RFRP-3	Downregulates GnRH expression; directly inhibits gonadotropin release			(4)
		NPFFR-1	Hyp, Thal, Amyg, Cb, Hpc, SC	NPFF		Potentially anorexigenic	Pro- and anti-opioid effects	(13)
**Marmoset** (*Callithrix jacchus*)		GPR147	Hyp	RFRP	Inhibits reproduction during the prepubertal period			(31)
**Pig **(*Sus scrofa*)	F	GPR147	Hyp, Pit, OB, MO, Cb, Cbr, Hpc, Ov, MO, SC, spleen, uterus, eye, adrenal, kidney, intestine	GnIH	Regulates the estrus cycle in sexually mature animals at all levels of the HPG axis			(32)
	F	GPR147	Hyp, Pit, Ov	RFRP-3	Inhibits GnRH; downregulates gonadotropin synthesis; downregulates estradiol secretion			(33)
		NPFFR-1	Hyp, Pit	RFRP-3	Suppresses LH pulses; regulates sexual maturation			(34)
**Cat **(*Felis catus*)	F	NPFFR-1	Ov	RFRP-3	Increases progesterone production from preantral follicles			(35)
**Rat **(*Rattus norvegicus*)		GPR147	Hyp	RFRP	Controls the prepubertal state and reproductive development			(36)
		NPFFR-1	PVN, mPOA, AHN, DMH, PMv, LS, Thal, Amyg	NPVF			Anti-opioid effects	(16)
		NPFFR-1		NPAF				
		NPFFR-1	Hyp, Pit, T, Ov, Thal, Amyg, OB, adrenal	NPFF		Potentially anorexigenic	Indirect role in the dopaminergic system; pro- and anti-opioid effects	(13)
		OT7T022	Hyp, Pit, T, Ov, Cbr, BG, Hpc, Thal, Mes, Cb, MO, SC, optic nerve, eye, adrenal, placenta	RFRP-1	Increases prolactin secretion			(15)
		OT7T022		RFRP-3				
	M	NPFF1R	Amyg	RFRP-1		Anorexigenic		(37)
		GPR147	RP3V, Arc, MS, POA, Pit, Hpc	RFRP-3	Regulates the central control of reproduction in adults			(38)
**Mouse** (*Mus musculus*)	M	NPFF1R	Hyp	RFRP-3		Orexigenic action likely *via* the modulation of the effects of leptin and ghrelin on feeding behavior; involved in the regulation of glucose homeostasis		(39)
	F	NPFF1R	Hyp	RFRP-3		No effect on feeding behavior; role in the homeostatic control of body weight and body composition in basal conditions; regulates energy expenditure		
		GPR147	DS	RFRP-3	Inhibits GnRH neurons			(40)
		GPR147	GnRH neurons, Kiss neurons, PVN, LS	RFRP-3	Inhibits Kiss and GnRH neurons			(24)
		GPR147	GnRH neurons, gonadotropes	GnIH				(41)
		GPR147	Gonadotropes	RFRP-1/3	Downregulates gene expression of LH-β, FSH-β, and common α- subunits in presence of GnRH stimulation; inhibits LH release			(42)
**AVES**								
Species	Sex	Receptor		Ligand	Reported Function			References
		Name	Localization		Reproductive	Metabolic	Other	
**Japanese quail **(*Coturnix japonica*)	M	GnIH-R	Pit, Cbr, Mes, SC	GnIH	Inhibits gonadotropin release; suppresses testosterone production and testicular development; negatively regulates the development of secondary sex characteristics			(5, 8)
		GnIH-R	Dien, Pit, Ov, T, epididymis, vas deferens, germ cells	GnIH	Downregulates reproduction at all levels of the HPG axis; regulates steroid synthesis and release, sperm maturation, and germ cell differentiation			(10)
**Chicken **(*Gallus gallus*)		RFRPR	Dien, Pit, Tel, OT, OB	GnIH	Regulates gonadotropin release			(7)
		NPFFR	Dien, Pit, Ov, T, Tel, OT, Cb, OB, MO, SC, eye, heart, liver, adrenal, spleen	GnIH				
		NPFFR-1	Hyp	GnIH				(43)
	F	GnIH-R	Dien, Pit	GnIH	Control of the prepubertal state; regulates the termination of reproduction			(44)
		GnIH-R	Hyp	GnIH		Orexigenic effects		(45)
		GnIH-R	T, Ov, prehierachiral follicles	GnIH	Possibly downregulates gonadal steroids; functions in follicular selection and maturation			(46)
**Turkey **(*Meleagris gallopavo*)	F	GnIH-R	Pit	GnIH	Reduces egg production efficiency			(47)
**European starling **(*Sturnus vulgaris*)		GnIH-R	Mes, PO region, GnRH-I neurons, GnRH-II neurons	GnIH	Inhibits the GnRH system			(18)
		GnIH-R	Dien, Pit, T, Ov, Mes, oviduct	GnIH	Downregulates reproduction at all levels of the HPG axis			(10)
**White crowned sparrow **(*Zonotrichia leucophrys*)	F	GnIH-R	Dien, GnRH-II neurons, ME, BNST, OMC	GnIH	Suppresses LH release; inhibits copulation solicitation behavior			(9)
		GnIH-R	Pit, Ov, T	GnIH	Downregulates reproduction at the level of the gonad			(10)
**House sparrow **(*Passer domesticus*)		GnIH-R	T	GnIH	Inhibits gonadotropin-induced testosterone secretion			(11)
TELEOSTS								
Species	Sex	Receptor		Ligand	Reported Function			References
		Name	Localization		Reproductive	Metabolic	Other	
**Goldfish **(*Carassius auratus*)		GnIH-R1*^†^	Hyp, PI of Pit, Thal, PeVN, NAT, NDTL, NDLI	GnIH	*Directly downregulates GnRH expression; ^†^Suppresses gonadotropin release			(48)
		GnIH-R2*^†^	Hyp, PI of Pit, Thal, POA	GnIH				
		GnIH-R3*	Hyp, Thal, POA	GnIH				
		GnIH-R1	Ov, T	GnIH	No effect in females; increases testosterone, upregulates StAR and 3βHSD, and downregulates CYP19 in males			(49)
		GnIH-R2	Ov, T	GnIH				
		GnIH-R	Pit	GnIH	Regulates gonadotropin release and mRNA expression of LH-β and FSH-β subunits; may be stimulatory or inhibitory to control seasonal reproduction			(50)
**Zebrafish **(*Danio rerio*)		GnIH-R1*	B, T, spleen, eye, muscle, kidney	GnIH	*Downregulates GnIH; potentially downregulates steroidogenesis and gametogenesis; ^†^Role in embryonic and early larval development; ^‡^Mediates the hypophysiotropic action of GnIH			(51)
		GnIH-R2*^†^	B, T, eye, kidney	GnIH				
		GnIH-R3*^‡^	B, Pit, T, Ov, spleen, eye, gill, muscle	GnIH				
		LPXRF-R2	Pit	LPXRFa-1/2/3	Downregulates LH β-subunit and CGA expression; no effect on FSH β-subunit expression			(52)
		LPXRF-R3	Pit	LPXRFa-1/2/3				
		NPFFR1-1	Dien, Pit, Tel, Mes, Rhom, OB	LPXRFa	Upregulates GnRH-3 expression in the Hyp and the FSH-β subunit in the Pit			(53)
		NPFFR1-2	Dien, Pit, Tel, Mes, Rhom	LPXRFa				
		NPFFR1-3	Dien, Pit, Tel, Mes, Rhom	LPXRFa				
**Common carp **(*Cyprinus carpio*)		GnIH-R1*	Hyp, T, Ov	GnIH-III	*Downregulates GnRH-3; ^†^Inhibits LH-β and FSH-β subunit expression			(54)
		GnIH-R2*^†^	Hyp, Pit, T, Ov	GnIH-III				
		GnIH-R3*^†^	Hyp, Pit, T, Ov	GnIH-III				
**Catla **(*Catla catla*)		GnIH-R1	B, G, kidney, liver, heart, gill, eye, stomach, intestine	GnIH	Primary site for GnIH action in the brain			(55)
		GnIH-R2	B, G, kidney, muscle, heart, eye, stomach	GnIH				
		GnIH-R3	G, kidney, liver, heart, gill, eye, intestine	GnIH				
**Nile tilapia **(*Oreochromis niloticus*)	F	LPXRF-R	Dien, Pit, T, Ov, Tel, Mes, liver, intestine, adipose, muscle, gill, heart, stomach	LPXRFa	Upregulates LH and FSH			(56)
**Orange-spotted grouper **(*Epinephelus coioides*)	H	GnIH-R	Hyp, Pit, G, OB, Tel, OT, Cb, MO, gill, kidney, stomach	GnIH-I/II/III	Decreases GnRH in brain and suppresses LH release			(19)
**Clownfish** (*Amphiprion melanopus*)	H	GnIH-R	B, Pit, G, eye	GnIH	Downregulates gonadotropins; potential role in sex change			(57)
		GnIH-R	Dien	GnIH	Downregulates GnRH expression and suppresses LH and FSH synthesis and release			(58)
**Grass puffer **(*Takifugu niphobles*)		LPXRFa-R	Dien, Pit, Tel, OT, eye	LPXRFa	Upregulates LH-β and FSH-β subunit expression			(59)
**Tongue sole** (*Cynoglossus semilaevis*)		LPXRFa-R	B, Pit, Ov, gill, heart, liver, spleen, kidney, stomach, intestine, muscle	LPXRAa-1	Stimulatory action			(60)
		LPXRFa-R	B, Pit, Ov, gill, heart, liver, spleen, kidney, stomach, intestine, muscle	LPXRAa-2	Inhibitory action			

^1^Sex abbreviations: M, Male; F, Female; H, Hermaphrodite. ^2^Localization abbreviations: Amyg, Amygdala; AHN, Anterior hypothalamic nucleus; Arc, Arcuate nucleus; BG, Basal ganglia; BNST, Bed nucleus of the stria terminalis; B, Brain; Cb, Cerebellum; Cbr, Cerebrum; Dien, Diencephalon; DS, Dorsal septal nucleus; DMH, Dorsomedial hypothalamus; G, Gonad; HbN, Habenular nuclei; Hpc, Hippocampus; Hyp, Hypothalamus; LS, Lateral septum; mPOA, Medial preoptic nucleus; MS, Medial septum; ME, Median eminence; MO, Medulla oblongata; Mes, Mesencephalon; NAT, Nucleus anterior tuberis; NDLI, Nucleus diffuses lobi inferioris; NDLT, Nucleus diffuses tori lateralis; BNST, Nucleus stria terminalis; OMC, Oculomotor complex; OB, Olfactory bulb; OT, Optic tectum; OV, Ovary; PVN, Paraventricular nucleus; PI, Pars intermedia; PT, Pars tuberalis; PeVN, Periventricular nucleus; Pit, Pituitary; POA, Preoptic area; Rhom, Rhombencephalon; RP3V, Rostral periventricular area of the third ventricle; SC, Spinal cord; SCN, Suprachiasmatic nucleus; SON, Supraoptic nucleus; Tel, Telencephalon; T, Testis; Thal, Thalamus; VMH, Ventromedial hypothalamus.

Like all GPCRs, GnIH-Rs are composed of seven interconnected transmembrane domains along with an N-terminal extracellular and a C-terminal intracellular tail. Following the original identification of the human GnIH-R, transfection studies in Chinese Hamster Ovary (CHO) cells revealed that activation of the receptor results in decreased forskolin-induced cAMP accumulation, while no effect on Ca^2+^ mediated signaling was observed, suggesting inhibition of adenylyl cyclase, and thus coupling to Gα_i_ or Gα_o_ (15). The specific inhibition of forskolin-induced cAMP accumulation in CHO cells transfected with the human GnIH-R was further confirmed by Mollereau et al. (62). Similarly, in chickens, initial studies suggested GnIH could modulate the levels of Gα_i2_ mRNA in COS-7 cells transiently transfected with the GnIH-R, suggesting activation of Gα_i_ (7). This was later confirmed *in vitro* as GnIH was shown to block forskolin-induced cAMP accumulation in GH_3_ cells (a rat pituitary somatolactotrope line) transiently transfected with the chicken receptor (44). In addition, in this study, co-transfection of the chicken GnIH-R and gonadotropin-releasing hormone receptor III (GnRH-RIII) showed that activation of the GnIH-R resulted in the reduction of GnRH-induced cAMP response in a receptor ratio-dependent manner, suggesting a direct interaction between the signaling of both GnIH-R and GnRH-RIII in chickens (44).

In fish, the intracellular signaling pathways used by GnIH-Rs have also been studied *in vitro* (for review: 63). Although, in tilapia, activation of the GnIH-R with LPXRFa-2 was shown to stimulate reporter constructs for both PKA and PKC suggesting coupling to Gα_s_ and Gα_q_ (56), stimulation of all three zebrafish GnIH-Rs (LPXRF-R1, LPXRF-R2 LPXRF-R3) transfected in COS-7 cells failed to activate reporter constructs for PKC while LPXRF-R2 and LPXRF-R3 exhibited a dose-response activation of reporter constructs for PKA, suggesting exclusive coupling to Gα_s_ (52). However, inhibition of cAMP accumulation was not measured in the above-mentioned studies. Further investigation in CHO cells demonstrated that both human NPFF-Rs (including GnIH-R or NPFF-R1) couple to Gα_i3_ and Gα_s_ as the primary transducers, with NPFF-R2, additionally coupled to Gα_i2_ and Gα (64). This suggests that ligand binding could result in opposing signaling pathways and may in part explain the conflicting results outlined in **Table 1**. Interestingly, studies in mammals showed that although GnIH orthologues are the preferential ligand for GnIH-R, other members of the RFamide family can also bind and activate it. For example, the human GnIH-R was shown to also bind several other endogenous peptides possessing an FLFQPQRFa sequence, although with lower affinity (62). In fact, cross-activation of receptors by multiple members of the RFamide family commonly occurs, as demonstrated by kisspeptin-10 and kisspeptin-54 shown to bind and activate both NPFF receptors, including the GnIH-R. In any case, this resulted in increased Ca^2+^ mobilization and decreased cAMP accumulation, confirming the coupling of mammalian GnIH-Rs to both Gα_q/11_ and Gα_i/o_ (65).

Interestingly, in the case of kisspeptin, the cross-activation appears to be unidirectional, as GnIH orthologues failed to significantly bind to and activate GPR54, the known receptor to kisspeptin (65). As RFamides and their receptors have significant clinical implications in humans, specific agonists and antagonists have been designed over the years. However, as for their native ligand, specificity may be an issue due to the promiscuity of NPFF-Rs (for review: 17). As a matter of fact, known agonists of GPR54 were shown to also bind to the GnIH-R and elicit intracellular Ca^2+^ mobilization, although they failed to mediate a decrease in cAMP accumulation (65). This suggests that while mammalian GnIH-Rs can couple to both Gα_q/11_ and Gα_i/o_, the activation of downstream signaling is dependent on the ligand. In fact, this lack of receptor subtype selectivity can be a significant challenge for the development of therapeutics, as recently discussed by Nguyen et al. (66).

Nonetheless, beyond therapeutic applications, the promiscuity of ligands for NPFF-Rs have significantly widened the physiological relevance of RF-amide peptides, including GnIH. For example, NPFF has a strong affinity for the GnIH-R (13), suggesting that the physiological impacts of GnIH-R activation depend on receptor localization and ligand availability. In mammals, particular attention has been placed on the role of NPFF and its receptors on modulating nociception, especially as it relates to opioid-induced analgesia (67, 68). Interestingly, the distribution of GnIH-R (NPFF-R1) and NPFF-R2 within the central nervous system differs amongst mammalian species (13), further highlighting the importance of both the presence of receptors and ligand availability. Nonetheless, the presence of GnIH-Rs throughout the hypothalamus suggests involvement in multiple neuroendocrine processes, and while GnIH was first identified as an inhibitory hypothalamic peptide on reproduction (1), it has since been shown to also participate in behavior, stress, and metabolism (for review: 3), all associated with energy balance and nutrient partitioning.

## GnIH and Its Receptor in the Brain, Involvement in Multiple Neuroendocrine Systems

Since its discovery, GnIH has been localized throughout the brain of many species, especially the diencephalon and mesencephalon, with particular emphasis on the hypothalamic region (4, 27, 69, 70). Despite some variations among species, GnIH perikarya have been located in the paraventricular nucleus (PVN) of quail (71, 72) and many other wild bird species (18, 73–75), the dorsomedial hypothalamic area (DMH) in hamsters and mice (14, 27), the periventricular nucleus (PerVN) in rats (15), and the dorsomedial nucleus, as well as the PVN in sheep (76). Neuronal projections have also been identified extending throughout the brain, including the preoptic area (POA), lateral septum, arcuate nucleus (ARC), and anterior hypothalamus in mammals (14, 70, 77, 78). Similar to the expression patterns of GnIH, GnIH-R is expressed throughout the hypothalamus, specifically in the POA, rostral periventricular area of the third ventricle (RP3V) and ARC. With further expression in the pituitary gland (38), this widespread localization within the hypothalamic area strongly suggests multiple neuroendocrine functions. However, the expression of GnIH and its receptor in the ARC is of particular interest, as this area is involved in the regulation of both reproduction and energy homeostasis (reviewed by: 79), which is further discussed below.

### Neuroendocrine Control of Reproduction

#### Seasonal Breeders

##### Long Day (LD) Breeders

Species such as chickens, deer, horses, and fish such as salmon, carp, seabass, tilapia, goldfish, and grass puffer (50, 59, 80–90), are diurnal seasonal breeders known to be reproductively active under long day lengths. Since the first species in which GnIH was identified was the quail (1), most of the early research on the effect of GnIH on reproduction involved avian species and the relationship with photoperiodicity. Specifically, an increase in melatonin released by the pineal gland and retina of the eye during the dark period results in an elevation of GnIH synthesis and release from the hypothalamus (86). In turn, binding of GnIH to its receptor on GnRH-containing neurons inhibits GnRH synthesis and release, while binding to its receptor in the anterior pituitary directly inhibits the production of gonadotropins (5). This inhibition of LH secretion was further confirmed in several other avian species, including chickens, white-crowned sparrows, and quail (5, 73, 91). As in avian species, the photoperiodic control of reproduction in many fish species is regulated by melatonin released from the retina of the eye and the pineal gland (Review by: 88), suggesting a common mechanism involving GnIH. As a matter of fact, administration of GnIH-3 downregulates GnRH and LHβ mRNA levels in goldfish (48), clownfish (57), zebrafish (52), and sole fish (92). Additionally, GnIH-3 administration decreases the expression of GnRH-I in the orange-spotted grouper (19), while in the common carp, expression of both LHβ and FSHβ are downregulated (54). Conversely, in the sockeye salmon, *in vitro* stimulation of pituitary cells with all three GnIH orthologs induced an elevation in FSH and LH release (93), while *in vitro* treatment of grasspuffer fish pituitary cells with goldfish GnIH resulted in an elevation in FSHβ and LHβ subunit mRNA levels (59). Interestingly, the inhibitory effects of GnIH on gonadotropins were observed upon *in vivo* treatment, while the stimulatory effects were obtained *in vitro* using primary pituitary cell cultures and would need to be further confirmed *in vivo*.

In avian species, upon photostimulation, decreasing levels of melatonin result in a decrease in GnIH synthesis, thus lifting the inhibition on the hypothalamic-pituitary-gonadal (**HPG**) axis and allowing for the release of GnRH and the subsequent activation of pituitary gonadotropes (94, 95). Interestingly, in chickens, once the axis has been activated, ovarian production of estradiol downregulates the expression of the GnIH-R in the pituitary gland (46), thus switching the sensitivity of the adenohypophysis in favor of stimulatory inputs (44, 94).

In nocturnal species, such as hamsters, studies on the role of GnIH frequently resulted in conflicting results, with both stimulatory and inhibitory effects reported (96, 97). This is possibly due to the contrasting role of melatonin in these species, with elevated levels during the dark phase contributing to heightened activity. In fact, central administration of GnIH stimulated the HPG axis of Syrian and Siberian male hamsters exposed to short day (SD) lengths, with GnIH triggering the release of LH (27, 98). However, when male Siberian hamsters were exposed to long day (LD) lengths, GnIH administration led to an inhibition of LH release (27). Additionally, gonadotropin production was suppressed in female Syrian hamster following intracerebroventricular (ICV) injection of GnIH, with no day length effect reported in this study (14). Therefore, additional sex-specific differences may be at play in this species as GnIH-R mRNA levels were reported to be higher in females than males across all tissues examined (23). However, GnIH-R mRNA levels were consistently elevated in hamsters maintained under LD versus SD, regardless of sex (23), suggesting differential regulation of GnIH and GnIH-R by melatonin and/or photoperiod compared to diurnal breeders. Nonetheless, in the same study, GnIH fibres were found to be more abundant in females under SD than LD, while no changes were observed in males (23). Interestingly, in ovariectomized Syrian hamsters, it has been suggested that time of day is critical in the determination of the sensitivity of the HPG axis to GnIH administration, with GnIH-R and LHβ mRNA levels exclusively downregulated when ICV injection occurred in the afternoon, while morning administration had no effect (97). This is consistent with the established model of diurnal, seasonal breeders and the timing of the LH surge as GnIH-ir cells significantly declined at the time of the surge in Syrian hamsters (25).

##### Short Day (SD) Breeders

In the case of short-day seasonal breeders such as the rhesus monkey, elevated melatonin levels lead to the advancement of puberty and the presentation of sexual cues (99). In this species, GnIH mRNA levels were upregulated in the pre-pubertal phase, and due to the association with GnRH during this period, it has been suggested that the inhibitory activity of GnIH contributes to the pulsatility and reduced firing rate of GnRH neurons, maintaining reproduction in an inactive state (31, 100). Furthermore, GnIH and its receptor were observed to be highest in adults (31). In sheep, GnIH treatment has also been shown to reduce the synthesis and release of gonadotropins (76, 101), and more recently, the use of a GnIH-R antagonist, RF9, resulted in the stimulation of gonadotropin production in ewes (102). However, upon further investigation, RF9 was not only found to be nonspecific to NPFF-R1 (GnIH-R) and NPFF-R2, but also acted as an agonist of GPR54, which could have resulted in the stimulation of LH release (103). To overcome the lack of specificity, additional antagonists were developed, with RF313 and GJ14 displaying moderate to high specificity for the GnIH-R with no impact on GPR54 (103, 104). In fact, while GJ14 does not impact forskolin-induced cAMP production, it was shown to block all effects of GnIH (103), making this receptor antagonist a valuable tool for future studies.

#### Non-Seasonal Breeders

Non-seasonal breeders include spontaneous ovulating species, such as humans, rats, mice, and naked mole rats, as well as induced ovulating species, such as the domestic cat. Unlike short-day and long-day breeders, the literature on non-seasonal breeders is more consistent, and GnIH and its receptor were shown to be present in all three levels of the HPG axis (13, 15, 30, 35, 105). Interestingly, puberty in humans has been hypothesized to be better anticipated by measuring the accumulation of fat rather than using age or environmental stimuli as a predictor (106). Therefore, it is not surprising that a direct role of GnIH on the hypothalamic control of reproduction appears to be substantially less critical than in seasonal breeders, as demonstrated by the reduced number of projections of GnIH neurons to GnRH neurons in the mouse (14) and GnIH projections to the median eminence (ME) in the rat and mouse (70, 77, 107). However, IV administration of GnIH still resulted in an inhibition of the GnRH-induced LH production in ovariectomized rats (107, 108). Although a decline in the expression of both GnIH and GnIH-R were reported around sexual maturation in male rats, while in females, the expression of GnIH increased and the expression of GnIH-R significantly declined between 28 and 49 days of age (36), again indicating a possible sex-related difference in the regulation of and sensitivity to GnIH. In naked mole rats, only dominant females can undergo spontaneous ovulation and breeding females display elevated numbers of kisspeptin cells while non-breeders presented an elevated number of GnIH cells (105). Beyond puberty and ovulation, GnIH levels in rats significantly declined immediately following parturition (109), likely playing a role in facilitating postpartum estrus in this species (110, 111). Thus, although the role of GnIH may be more discrete in non-seasonal breeders, the inhibitory impact on reproduction is still conserved.

#### Interaction With Kisspeptin and Its Receptor

Since its initial discovery as a novel gene in humans (112), kisspeptin, along with its receptor (GPR54/Kiss-1r; 113), was shown to control puberty and reproduction in mammals *via* direct stimulation of GnRH neurons (114–119). In fact, along with neurokinin B and dynorphin, kisspeptin is part of an intricate neuronal circuitry responsible for the pulsatile secretion of GnRH, referred to as the KNDy neurons (for review: 120). Interestingly, GnIH-Rs are expressed in 9-16% of RP3V kisspeptin neurons in rats (24), as well as in 5-10% of the anteroventral periventricular nucleus (AVPV) and 25% of ARC Kiss1 neurons in mice (121). Additionally, Kiss1 neurons in the ARC are in close proximity to GnIH fibres (121), suggesting that GnIH may directly inhibit a subset of kisspeptin neurons and thus inhibit reproduction (122), although a reciprocal effect was not identified (121). In mice, GnIH-R knockout (KO) resulted in a weaker disruption of LH secretion (123) compared to GPR54 KO (124). However, *Kiss1* mRNA was found to increase in GnIH-R KO mice (123), and with 33% of GnRH neurons also expressing GnIH-R in rats, GnIH likely acts at multiple levels (GnRH and kisspeptin neurons) to inhibit GnRH synthesis and release (24). This is further supported by the similarities in expression patterns between GnIH-R and GPR54 as previously reported (15, 125).

In long-day breeders, shorter day lengths result in increased expression of GnIH due to higher melatonin (86), while the number of kisspeptin-positive cells in the ARC and *Kiss1* mRNA levels decline (126–129). Interestingly, when hamsters maintained under a short-day length were treated with Kp10, maturation of the reproductive tract was observed, with organ weights comparable to that under long days (96), indicating that exogenous kisspeptin is able to override the need for photostimulation. As discussed further in the following sections, beyond photoperiod, switching the activity of the HPG axis from inhibitory to stimulatory may intimately be linked to metabolic status and the hypothalamic control of energy reserves and nutrient partitioning (130, 131). This is further supported as a relationship between kisspeptin, photoperiod, and food availability has previously been established in seasonal mammals (132).

Furthermore, as avian species require a significant shift in energy partitioning towards egg production, metabolic status may be the primary cue controlling the activation of the HPG axis (133). Thus, the absence of a Kp gene in several avian species (134, 135) may allow GnIH to take a central role in balancing energy status and reproduction.

### Integration With the Central Regulation of Metabolic Processes

#### Regulation of Body Weight and Composition

The relationship between reproduction and body weight has been previously described in mammalian and avian species (133, 136–139), with a minimum body weight threshold required to initiate the activation of the HPG axis (140, 141). Prior to sexual maturation, animals undergo a rapid weight gain and growth phase while the HPG axis remains suppressed, possibly *via* elevated levels of GnIH. For example, moderate to high intraperitoneal doses of GnIH in mice were reported to evoke an increase in body mass (142), and chronic GnIH ICV injection elevated both body weight and feed intake in male mice (143). Initially, it was proposed that body weight was under the dual control of both GnIH and its stimulatory counterpart, as GnRH agonist treatment also resulted in a dose-dependent body mass gain in rats (144). However, the impact GnRH had on body weight was likely the outcome of a GnRH-stimulated increase in the expression of neuropeptide Y (NPY), as this peptide not only stimulates feed intake but has additionally been implicated in the preovulatory surge (145, 146). Furthermore, as described in the following section, more recent evidence suggests that beyond the central nervous system, GnIH can also influence body weight *via* direct control of adiposity in male mice, acting independently of reproductive steroids or the melanocortin system (96).

In view of the promiscuity between ligand and receptors from the RFamide peptide family, it is reasonable to question whether the impact of GnIH on body weight is mediated through its own receptor. This could partly be answered using GnIH-R KO, and in mice, inactivation of the GnIH-R resulted in significantly heavier females, yet it did not impact the weight of males (39), suggesting that the impact of GnIH on body weight is sex-dependent and most likely related to reproduction. Since in mammals, the GnIH-R was shown to couple to both Gα_i3_ and Gα_s_ (64), the observed differences between males and females may point to a differential receptor activation and signalling between sexes (147). In fact, when GnIH-KO and control mice were fed high-fat diets (HFD) and low-fat diets (LFD), males displayed declined locomotor activity in both KO groups, while female mice in both KO groups demonstrated elevated fat mass over the control (39). However, regardless of sex, obese (ob/ob) mice displayed lower GnIH mRNA levels in the dorsal-medial nucleus than their wild-type counterparts (148). Taken together, these studies suggest that as proposed by Cázarez-Márquez et al. (96), the impact of GnIH and its receptor on body weight may be linked to adiposity, which is also intimately associated with reproduction. Back in 1974, Frisch and McArthur (106) postulated the ‘critical weight hypothesis,’ which states that the accumulation of body fat stores may be a better indicator of the timing of sexual maturation rather than age or body weight itself. This has further been confirmed in rats, as well as chickens, with insufficient fat stores resulting in pubertal delay (136, 149, 150) and diet-induced obesity resulting in declined reproductive capacity (151, 152). In a recent study, GnIH injections resulted in the elevation of serum total triglycerides and cholesterol (153), leading to increased uptake of triglycerides by the adipose tissue (142). Thus, it could be hypothesized that GnIH acts to control fat deposition in adipose tissue not only in immature animals but also in adults. This is partially supported by the association between GnIH-induced increased body weight and increased brown adipose tissue (BAT) mass and liver mass (143). However, although GnIH appears to stimulate fat deposition, male GnIH-R KO mice were not protected from body weight gain on a high-fat diet (39), and a lack of GnIH-R signalling did not prevent obesity. Additionally, with GnIH-stimulated feed intake reported to trigger dyslipidemia (153) and abnormal glucose metabolism (143, 154), GnIH may play a larger peripheral role in the control of metabolism than previously thought (discussed in a following section).

#### Regulation of Feed Intake *via* the Melanocortin System

It is well-established that the melanocortin system controls feed intake through the orexigenic peptides, neuropeptide Y (NPY) and agouti-related peptide (AgRP), and the anorexigenic peptides, pro-opiomelanocortin (POMC) and cocaine-and-amphetamine regulated transcript (CART) (155–160). Neurons from the melanocortin system have been localized throughout many regions of the hypothalamus (24, 161) and shown to be in close contact with GnIH neurons in the DMH, with GnIH-containing fibres projecting to the ARC and PVN in mice (77, 162). In fact, the orexigenic effect of GnIH is mediated through the modulation of POMC and NPY neuronal activity (24, 161, 163, 164), with POMC downregulated in the presence of GnIH (161, 164, 165). In GnIH-R KO male mice, POMC mRNA levels in the hypothalamus are elevated compared to wild type (39), further suggesting a direct role for GnIH and its receptor in the inhibition of the anorexigenic response. However, the direct role of GnIH on NPY appears to be more ambiguous (161, 164). In mice, GnIH has been reported to inhibit the neuronal activation of NPY (161), yet in chicks, sheep, and rats, GnIH was shown to stimulate NPY, prompting an increase in feed intake (165, 166). Nonetheless, whether acting on both branches of the melanocortin system or not, ICV administration of GnIH increases feeding duration (167) and overall feed intake in chickens and rats (70, 108, 165, 168). However, the injection of GnIH into the amygdala of rats also resulted in an opposite effect with the suppression of food intake (37). Interestingly, despite mediating increases in feed intake in sheep, GnIH administration did not result in a reduction in energy expenditure, as measured by calorimetry (166). This strongly suggests that the effect of GnIH on feed intake is not intended to achieve or restore energy homeostasis but may rather coordinate the partitioning of energy away from reproduction (169). However, chronic ICV injection of GnIH in mice did decrease energy expenditure and increased feed intake, contributing to a rapid decline in brown adipose tissues (BAT) activity prior to an accumulation in lipid droplets, thus elevating BAT deposition overall (143). This effect also resulted in a GnIH-induced decrease in core body temperature during short exposure to the dark phase, suggesting for the first time that GnIH contributes to the conservation of energy (143).

In songbirds and zebra finches, food deprivation results in a decline in the number of GnIH-ir cells (170), although no differences in mRNA or peptide were observed (171, 172). As hypothesized by Fraley et al. (167), this decrease in GnIH-ir cells following feed restriction is likely the result of chronic metabolic stress. Of interest, declining GnIH-ir cell numbers have been correlated to declining body mass in female songbirds (170). When GnIH-R deficient male mice underwent 12-h fasting, despite a large decline in body mass, no change in LH secretion was detected. Conversely, in their wild-type counterparts, an immediate decline in LH secretion occurred following a 12-h fasting (123). Taken together, these studies provide an alternative pathway by which GnIH is able to mediate feed-seeking behavior *via* body mass fluctuations (170).

Since the leptin receptor is co-expressed with NPY and POMC, leptin is also thought to regulate feed intake (173, 174). Thus, a possible integration between GnIH and leptin has been investigated. This led to the identification of the long form of the leptin receptor (LepRb), present on 15 to 20% of GnIH neurons (148). This finding highlights a possible pathway between GnIH and the regulation of adiposity *via* leptin and feed intake (175). With diminished levels of GnIH observed in the leptin-deficient ob/ob mice, a direct inhibition from leptin *via* the small subset of LepRb-expressing neurons is possible, yet alternative indirect hypotheses have also been proposed (148). However, leptin acts through the PKC-dependent pathway to promote intracellular Ca^2+^ signalling within GnIH neurons, thereby potentially eliciting an indirect effect on appetite control and an indirect negative feedback to GnRH neurons (176), which lack the LepRb (177). Thus, elevated circulating leptin results in a decline in GnIH activity (178), leading to low LH levels in ob/ob mice, resulting in infertility (179). However, reactivation of the HPG axis is possible with leptin treatment (180, 181), illustrating a possible multipronged link between metabolism and reproductive success (182, 183), with leptin additionally proposed as a regulator in the timing of puberty (184). Furthermore, as both leptin and GnIH activate the PI3K/Akt signalling pathway (185, 186), Anjum et al. (175) hypothesized that PI3K/Akt signalling in ventromedial nucleus of the hypothalamus (VHM) neurons is responsible for the coordination of energy homeostasis (further discussed in a following section).

#### GnIH and Central Ghrelin

In addition to the melanocortin system, ghrelin, a hormone traditionally referred to as the ‘hunger hormone,’ is also involved in the control of appetite. As for GnIH, ghrelin elicits a stimulatory effect on food intake and an inhibitory effect on gonadotropin secretion through the growth hormone secretagogue receptor (GHS-R) (187) present on GnRH neurons (188). While this hormone has been primarily linked to the gastrointestinal tract, it was also identified in the brain of chickens (189), mice, and rats (190, 191), pointing to a neuroendocrine role beyond the gut. Specifically, ghrelin-containing hypothalamic neurons have been proposed to interact with NPY and AgRP-containing neurons to stimulate the secretion of orexigenic peptides in rodents (190, 191). Beyond appetite control, ghrelin can also inhibit LH (192), and testosterone production through the inhibition of steroidogenic enzymes (193), contributing to the suppression of the HPG axis during periods of insufficient energy stores (reviewed by: 194). Interestingly, when fed a HFD, GnIH-R KO mice demonstrated a complete resistance to the central administration of ghrelin, failing to prompt an increased cumulative feed intake compared to their wild-type counterparts (39). This suggests that the effect of ghrelin on appetite control may be in part mediated *via* GnIH and its receptor. However, the suppression of ghrelin effects in GnIH-R KO was not sustained when mice were fed a LFD (39). Since it has previously been reported that rats fed a HFD do not display ghrelin-induced hyperphagia but do maintain the increased adiposity also observed in LFD-fed rats, ghrelin may utilize separate mechanisms to act on appetite and lipid metabolism (195). In fact, Anjum et al. (175) suggested that the ghrelin receptor involved in the brain may be different and not yet identified.

### Integration With the Stress Response

The relationship between the hypothalamo-pituitary-adrenal (HPA) axis and GnIH has been previously established (Reviewed by: 196). GnIH neurons in the PVN are in direct contact with neurons containing corticotrophin releasing hormone (CRH); (163), which upon release, triggers the activation of the stress axis (197, 198). Furthermore, the CRH receptor 1 is expressed in 13% of GnIH neurons and its activation has been shown to elevate GnIH-R mRNA *in vitro* (199). As well, GnIH neurons have been shown to respond to mediators of acute and chronic stress (199–201). These stressors include but are not limited to, immunological stress, physical restraint, and social isolation and defeat. Specifically, a lipopolysaccharide (LPS) challenge in female rats resulted in a significant elevation in GnIH and GnIH-R expression, concurrent with a direct downregulation in LH-β mRNA (202). A similar inhibitory effect on the HPG axis was observed during social defeat stress in tilapia, with pituitary GnIH-R levels significantly increasing along with cortisol levels (203, 204). This was further validated in immobilized Wistar rodents (199), although in socially isolated male Sprague-Dawley rats, both GnRH mRNA expression and GnIH neuronal activity were suppressed, while no changes in the number of GnIH cells were detected (205). Taken together, experimental evidence suggests that CRH can directly activate a proportion of hypothalamic GnIH neurons and increase GnIH sensitivity by upregulating the GnIH-R. As for most neuroendocrine responses, chronic activation of the CRH receptor can lead to the desensitization of the HPA axis (206). Thus, it has been hypothesized that CRH sensitive GnIH cells may also become unresponsive, resulting in the interruption of the GnIH-GnRH neuronal pathway during sustained chronic stress (205). Since the GnIH-R is also expressed in pituitary corticotropes along with POMC, the precursor peptide of ACTH (207), the interactions between GnIH and the HPA axis appears bidirectional and in fact, ICV injection of GnIH in tilapia led to an elevation in plasma ACTH levels (208). However, recent studies have demonstrated that this is unlikely due to co-expression of GnIH-R and POMC, as pituitary expression of GnIH-R was elevated during social defeat, along with an elevation in GnIH mRNA and cell numbers, while POMC remained unaffected (204).

As discussed in the previous section, GnIH is implicated in metabolic control *via* feed intake and energy partitioning. In birds, metabolic stress can be induced by feed deprivation, resulting in serum corticosterone levels 13 times higher than under *ad libitum* feeding, although no change in the number of CRH-ir neurons were observed in the PVN (170). A positive correlation between serum corticosterone levels and mass loss was previously identified (170). However, whether the loss of mass was directly induced by corticosterone, or the consequence of reduced feed intake, is not clear and could potentially involve GnIH. Of interest, glucocorticoid receptors (GRs) are present in GnIH cells (201), and activation of GR *via* the administration of synthetic glucocorticoids such as dexamethasone stimulates GnIH and GnIH-R transcription (209). Interestingly, although chronic administration of corticosterone resulted in the stimulation of GnIH synthesis in the diencephalon of quail, acute injections did not (201), suggesting that the impact of stress on reproduction *via* GnIH is intended to adapt to longer-term impact. Furthermore, since GnIH is more effective at interrupting reproduction during the early breeding season in rock doves (210), the impact of stress on GnIH may allow the delay of sexual maturation when conditions, such as food availability, are not favourable.

### Integration With the Central Control of Thyroid Hormones

Similar to GnIH, THs have been implicated in both reproduction and metabolism. Since THs upregulate the basal metabolic rate of most cells of the body, hypothyroidism is associated with significant increases in body weight, along with high levels of TSH due to the absence of negative feedback (211, 212). Conversely, systemic or central (ICV) administration of T_3_ results in weight loss with a reduction in thyroid-releasing hormone (TRH) and TSH (213). These fluctuations in weight appear to also be related to observed fluctuations in GnIH, with changes in circulating TH concentrations inducing the inverse expression of GnIH (122). However, to the best of our knowledge, a direct effect of GnIH on the thyroid gland is not known, and the expression of the GnIH-R in follicular cells has not been reported. Nonetheless, it is well documented that hyperthyroidism ultimately leads to a net fat loss through an elevation in resting energy expenditure, decreased cholesterol levels, and increased lipolysis and gluconeogenesis (reviewed by: 214, 215), which could lead to reproductive dysfunction. Under fasting conditions, a decline in pituitary Dio2 levels was observed, leading to an inactivation of the HPG axis (216). Simultaneously, hypothalamic Dio2 levels were found to increase (216), resulting in an elevation in NPY and AgRP in the ARC and the inhibition of TRH production in the PVN (217). This likely occurs to defend energy stores (218, 219), stimulating feed intake to restore homeostasis. In fact, administration of leptin and alpha-melanocortin-stimulating hormone (α-MSH) restored TRH levels to normal following the fasting-induced decline (220–223). As leptin has been found to be correlated with body mass index (BMI) and TSH levels (224), this adipokine inhibits the orexigenic branch of the melanocortin system while stimulating POMC (222, 225). These stimulatory effects of leptin on TRH occur in both a direct mechanism in the PVN and the indirect mechanism in the ARC, acting through the melanocortin system (222). Intriguingly, in Dio2-KO mice, post-fasting feeding behavior did not return to normal levels (217), suggesting that T_3_ activation of the orexigenic peptides may be more critical than GnIH in restoring this behavior.

As elevated TH levels are known to activate GnRH neurons and indirectly suppress the activity of GnIH (226–228), it is unsurprising that GnIH mRNA levels decline with the administration of T_4_ and increase during periods of low circulating levels (122). In fact, GnIH-KO prevented the delay in pubertal onset typically associated with hypothyroidism (122). Furthermore, as is discussed in more detail in the following section, while GnIH has been shown to decrease insulin production, TH are known to exert an opposite role (229), leading to alterations in leptin concentrations. Thus, we hypothesize that leptin may be indirectly downregulated in the presence of GnIH, and therefore the re-activation of the HPT axis *via* leptin depends on the downregulation of GnIH observed under fasting conditions.

## Beyond Neuroendocrine Functions, GnIH and Its Receptor Participate in the Peripheral Regulation of Physiological Processes

### Peripheral Control of Reproduction

Although it is clear that GnIH and its receptor are key to the hypothalamic control of reproduction, the presence of the GnIH-R at the lower level of the HPG axis (gonads) has also been reported in avian species, including the Japanese quail, the European starling, the white-crowned sparrow (10), and the domestic chicken (7, 230), as well as various fish species including clownfish (57), Nile tilapia (56), Indian carp (61), common carp (54), zebrafish (51), and goldfish (49), and the rat (13, 15). Additionally, the GnIH-R has been identified in the ovary of humans (30), swine (33), felines (35), tongue sole (60), and in the testis of Syrian hamsters (26) and house sparrows (11) (**Table 1**). Furthermore, treatment with GnIH was shown to effectively shut down reproduction in the ovary and testis not only by indirectly reducing gonadotropins release from the pituitary as previously discussed, but also directly by decreasing cell viability in the ovary and reducing the levels of testosterone in the testis (5, 8, 10, 11, 46, 51). In fact, the GnIH-R has been found to play a role in the downregulation of steroidogenesis (7, 10, 11, 30, 46, 51). In humans, GnIH has been shown to downregulate the production of steroidogenic acute regulatory (StAR) protein, while the GnIH-R antagonist (RF9) was able to partly block this effect (30). Beyond steroidogenic enzymes, by suppressing glucose uptake (to be discussed in the following section), which normally promotes cholesterol uptake and thus metabolic substrates to germ cells (231, 232), GnIH may also reduce substrate availability resulting in a decline in sex steroid production in mammals (142). In turn, the inhibitory effect of GnIH produced in the gonads results in a decline in spermatogenesis (10, 26) or a decline in the viability of pre-hierarchical follicles leading to impaired follicular maturation (46). Similar to the anterior pituitary, treatment with estradiol and/or progesterone also downregulates the expression of the GnIH-R in the chicken ovary (46). Thus, the impact of GnIH on the inhibition of the HPG axis may become less influential once the animal reaches sexual maturity.

### Peripheral Metabolic Control

Similar to reproduction, recent evidence also points to a broader role for GnIH on peripheral tissues, especially as it relates to energy storage and availability. Recently, high levels of GnIH mRNA have been confirmed in the eye, while low levels were observed in the colon, stomach, ileum, muscle, kidney, and spleen (162). Interestingly, the expression pattern of GnIH in these peripheral tissues was similar to that of its receptor (162). Nonetheless, since this is a relatively new field of research, most studies described in the following section are based on the peripheral administration of exogenous GnIH rather than on the activity of endogenous GnIH itself.

#### Adipose Tissue

Although GnIH may not be produced in the adipose tissue, its receptor was shown to be present on human fat cells, suggesting a direct role for GnIH or its orthologues on adiposity (29). Interestingly, the initial focus was placed on white adipose tissue (WAT) due to its association with energy storage (233). However, it was recently reported that GnIH treatment inhibits the activity of BAT (143), which plays a critical role in energy expenditure through thermogenesis (reviewed by: 234, 235).

In terms of WAT, circulating leptin concentrations can be used as an indirect measure of fat accumulation (96, 236–238). For example, higher leptin concentrations are observed in fat or *ad libitum* fed Syrian hamsters compared to their respective lean or feed-restricted counterparts (239). Of interest, leptin is known to reduce feed intake (240) and is considered anorexigenic (241). This activity opposes the role of GnIH on appetite control discussed earlier and highlights a possible antagonistic effect between leptin and GnIH (178). This relationship is further emphasized in ob/ob mice, which lack a functional leptin gene (181). Without this WAT regulator, rats demonstrated an elevation in body weight (242, 243), similar to that observed in GnIH treated mice (143).

As described previously, both leptin and GnIH activate the PI3K/Akt signalling pathway in the hypothalamus, a pathway also used during insulin-mediated glucose uptake in the adipose tissue (142), thus, raising the possibility of an interaction between, GnIH, leptin, and insulin in the regulation of glucose uptake. Since GnIH-treated male hamsters had higher levels of leptin and insulin compared to controls (96), it is possible that this resulted in increased adiposity (142), leading to increased insulin resistance (reviewed by: 244). However, this effect was not observed in female hamsters (96). In fact, it appears that the effect of GnIH may be dose-dependent as 2,000 and 20 ng induced a decline and increase in glucose levels, respectively, with a concomitant elevation in insulin receptor and GLUT8 proteins under the lowest GnIH dose only (142). Thus, GnIH may regulate insulin sensitivity, at least in male mice. As the study by Cázarez-Marquez et al. (96) used significantly higher GnIH levels (105-μg), a possible GnIH threshold may exist with supraphysiological doses eliciting opposite effects.

#### Pancreas

As described in the previous section, GnIH has been hypothesized to regulate fat accumulation in adipose tissue through the mediation of increased nutrient uptake, including glucose and triglycerides (142). In fact, when injected intraperitoneally, GnIH has been found to primarily co-localize with glucagon in α-cells, while its receptor, although present in both cell types, is primarily co-localized with insulin in β-cells of pancreatic islets (153), strongly suggesting an involvement in glucose homeostasis. Essentially, GnIH supports α-cell survival and hyperplasia through activation of the GnIH-R present in these cells, which triggers the AKT and ERK1/2 pathways (186), leading to an increase in glucagon and feed intake. When glycemia is elevated, β-cells would normally release insulin to offset the imbalance, restore glucose homeostasis, and reduce feed intake (reviewed by: 245). However, recent evidence demonstrated that chronic and acute doses of GnIH increase blood glucose levels while simultaneously reducing insulin secretion. With the colocalization of GnIH and glucagon, it is likely GnIH can promote hyperglycemia in rats (153, 246). The direct blockade of insulin has been hypothesized to occur through Gα_i_ and the inhibition of the AC-cAMP-PKA pathway (153, 247). This hypothesis is further supported by the reduced insulin sensitivity with chronic GnIH treatment, characterized by a decline in insulin receptor and GLUT4 in the adipose tissue (142, 153). Intriguingly, since insulin is able to relay information regarding body fat status to the central nervous system (reviewed by: 248, 249), inhibition *via* GnIH further supports a multipronged impact through which GnIH promotes fat accumulation and an elevation in body weight, associated with increased feed intake (see previous section). Furthermore, kisspeptin has also been reported to stimulate glucose production with an increased glucose tolerance to prevent the onset of hyperglycemic disorders (250) *via* its receptor. As this effect is absent in GPR54-KO mice (251), it is possible that cross-activation by various members of the RFamide family, including GnIH, can occur. This is of particular interest as insulin has been reported to be a mediator between nutritional status and reproductive success (252), and significant fluctuations in glucose levels can be lethal (reviewed by: 253).

## Conclusion

**Figure 1:**
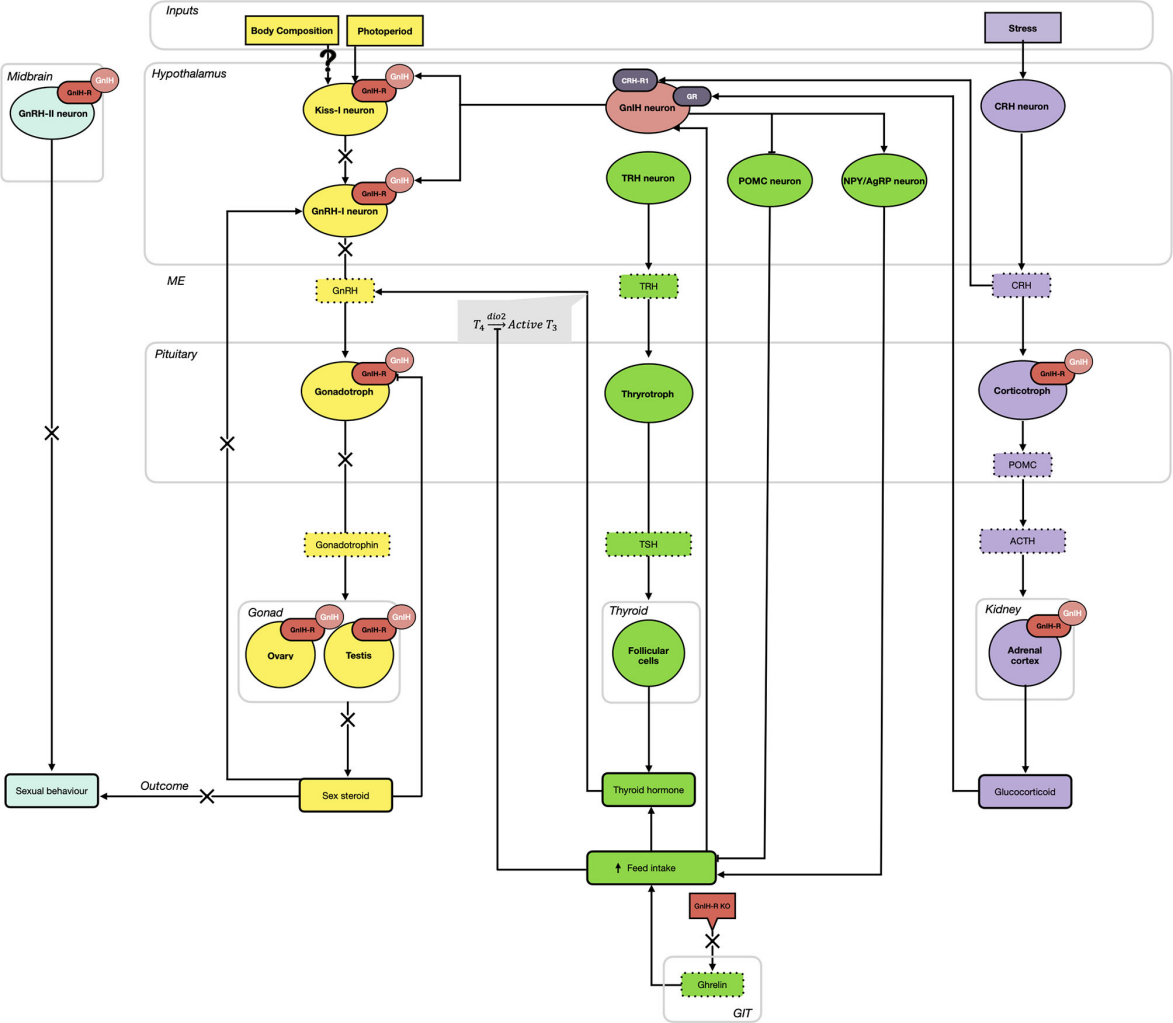
The hypothalamo-pituitary-gonadal (HPG; yellow), thyroid/metabolic (HPT; green), and adrenal (HPA; purple) axes are highly integrated *via* the activity of gonadotropin-inhibitory hormone (GnIH) and its receptor (GnIH-R). As part of the HPG axis, GnIH neurons elicit an inhibitory effect on gonadotropin-releasing hormone I (GnRH-I) neurons (hypothalamus) and gonadotroph cells (pituitary gland) in all species, in addition to suppressing the activity of kisspeptin (Kiss-1) neurons in mammalian species (hypothalamus). Additional GnIH-Rs on GnRH-II neurons in the midbrain contribute to the inhibition of sexual behaviors. In seasonal breeders, photoperiod influences the expression of GnIH, with short day (SD) lengths elevating GnIH and inhibiting reproduction, while long day (LD) lengths diminish GnIH expression and permits the progression of sexual maturation, *via* gonadotropin production. Acting at the level of the gonads (ovary and testis), the production of sex steroids further downregulates GnIH-R expressed in the pituitary gland. Simultaneously, GnIH neurons stimulate the orexigenic peptides, neuropeptide Y (NPY) and agouti-related peptide (AgRP), and downregulate the anorexigenic peptide pro-opiomelanocortin (POMC). Overall, this results in an increase in feed intake, promoting an upregulation of the HPT axis. Ghrelin from the gastrointestinal tract (GIT) also contributes to this elevation in feed intake and knockout (KO) of the GnIH-R can downregulate this pathway. Active conversion of thyroid hormones (T_4_ → T_3_) stimulates the release of GnRH from the hypothalamus, thus activating the HPG axis. Finally, the HPA (stress) axis also provides input to GnIH neurons. While GnIH can bind to pituitary corticotropes and the adrenal cortex to promote an elevation in adrenocorticotropic hormone (ACTH) and glucocorticoids, respectively, elevations in glucocorticoids positively feedback on GnIH neurons. This results in an inhibitory effect on reproductive activity, hypothesized to shift resources away from this energetically expensive process and towards managing the stressor.

**Figure 2:**
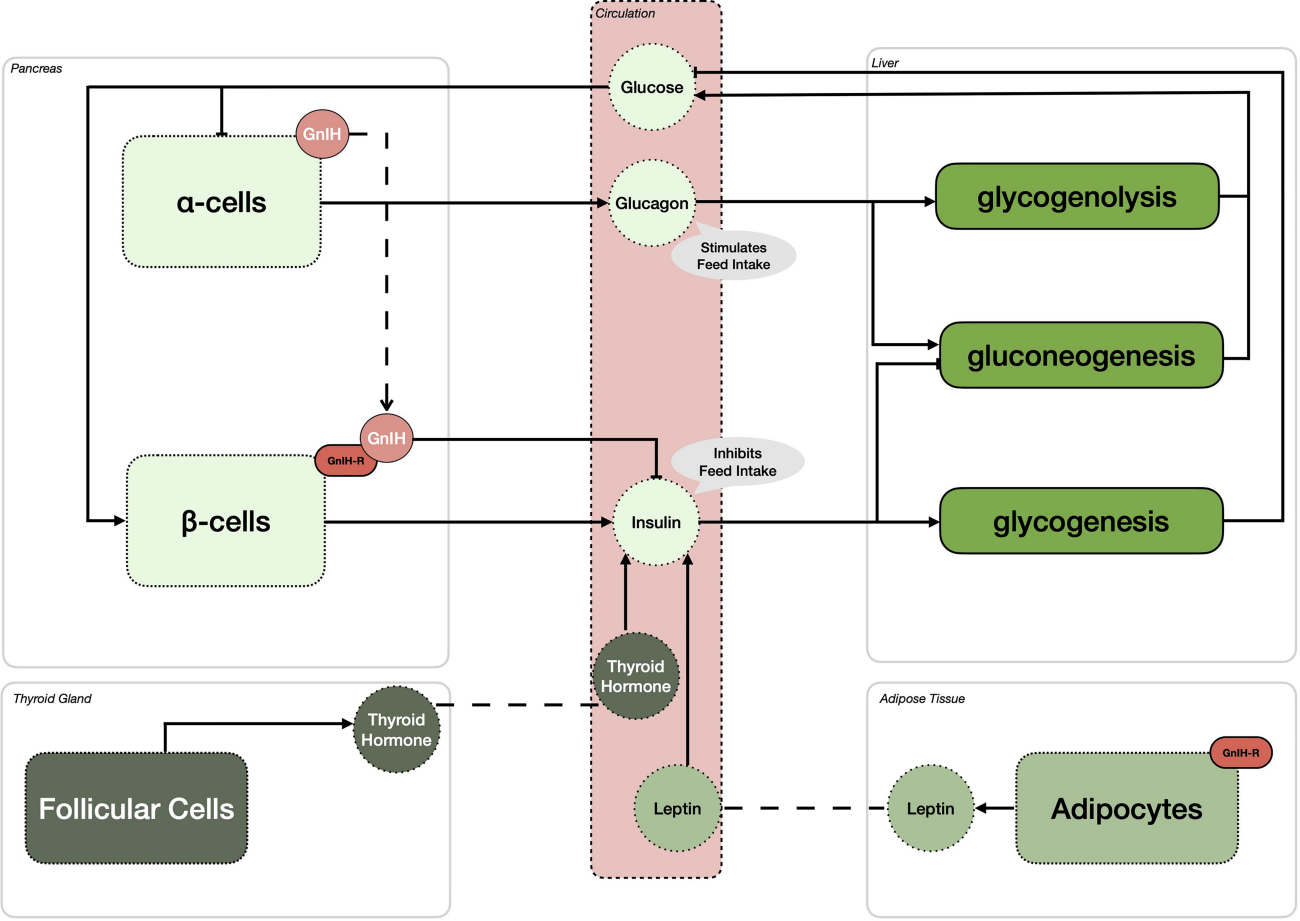
Gonadotropin-inhibitory hormone (GnIH) and its receptor (GnIH-R) impact metabolic control and energy partitioning in various peripheral organs. In the pancreas, GnIH is expressed in alpha cells (α-cells), while GnIH-R is expressed in the beta cells (β-cells). It is hypothesized that GnIH binding to its receptor will inhibit the production of insulin in β-cells, thus stimulating feed intake. In an effort to achieve homeostasis, high glucose levels stimulate the production of insulin leading to the stimulation of glycogenesis and inhibition of gluconeogenesis in the liver. However, in periods of low glucose availability, α-cells increase their production of glucagon, leading to the stimulation of feed intake, similar to the activity of GnIH. In addition, glucagon stimulates both glycogenolysis and gluconeogenesis, elevating the circulating levels of glucose. While the expression and presence of GnIH has yet to be reported in adipocytes, GnIH-R is. Thus, it is hypothesized that GnIH can influence circulating leptin concentrations, an hormone known to oppose the action of GnIH on insulin, thereby downregulating feed intake. As for leptin, thyroid hormones also stimulate the anorexigenic effect of insulin, thus counteracting the effect of GnIH on glucose mediated feed intake.

Since its initial discovery over 20 years ago, the roles and importance of GnIH and its receptor have significantly expanded. As shown in **Figure 1**, GnIH, *via* its receptor, not only directly inhibits the synthesis and release of GnRH and gonadotropins but also participates in the integration of multiple internal and external cues to control reproduction. Specifically, we propose that GnIH is responsible for modulating body composition and energy status and thus partitioning nutrients away from reproduction. This is achieved in part by stimulating feed intake *via* the melanocortin system, which in turn inhibits the induced release of GnRH in the ME by THs. Furthermore, we also propose that the negative impact of stress on reproduction is mediated in part *via* GnIH, as it is upregulated by both hypothalamic CRH and circulating glucocorticoids while also stimulating the expression of POMC in pituitary corticotrophs and glucocorticoids in the adrenal cortex. This results in a complex central integration between the HPG, HPT and HPA axes to allow or preclude reproduction from proceeding (**Figure 1**). In addition, recent evidence also suggests that GnIH and its receptor participate in the regulation of peripheral metabolic processes (**Figure 2**). In the pancreatic islets, GnIH produced by α-cells acts in a paracrine manner on its receptor present on β-cells to inhibit the production of insulin, thus counteracting the effect of THs and leptin and promoting feed intake while reducing gluconeogenesis and glycogenesis in the liver (**Figure 2**). Interestingly, the presence of the GnIH-R in adipose tissue suggests a more complex role on energy partitioning, requiring further investigation (**Figure 2**). Nonetheless, with the known promiscuity between members of the RF-amide family and their receptors, it is also possible that alternative ligands and receptors are also involved, opening new avenues for potential therapeutic applications.

Ultimately, while our understanding of the individual physiological roles played by GnIH and its receptor has been extensively investigated over the last two decades, integrative functions both centrally and peripherally are relatively recent and open the avenue to a new era of research.

## Author Contributions

GB is the lead author who coordinated the contributions of co-authors, elaborated the structure of the manuscript and wrote the main section related to reproduction. CH contributed to all sections of the manuscript with particular emphasis on metabolic control. CH also designed the figures and tables. KT was the architect of this review and provided the information as it pertains to the comparative aspect of this review. All authors contributed to the article and approved the submitted version.

## Conflict of Interest

The authors declare that the research was conducted in the absence of any commercial or financial relationships that could be construed as a potential conflict of interest.

## Publisher’s Note

All claims expressed in this article are solely those of the authors and do not necessarily represent those of their affiliated organizations, or those of the publisher, the editors and the reviewers. Any product that may be evaluated in this article, or claim that may be made by its manufacturer, is not guaranteed or endorsed by the publisher.
